# Supplementary material for: Novel Driver Strength Index highlights important cancer genes in TCGA PanCanAtlas patients
Source: PeerJ. 2022 Aug 11;10:e13860. doi: 10.7717/peerj.13860 (PMC9375969; doi:10.7717/peerj.13860)
Supplement: Supplemental Information 2 [file peerj-10-13860-s002.zip › COHORTS/genes plots/age/2021_8_16_14_9_distribution_cohorts_females_80-84.pdf]

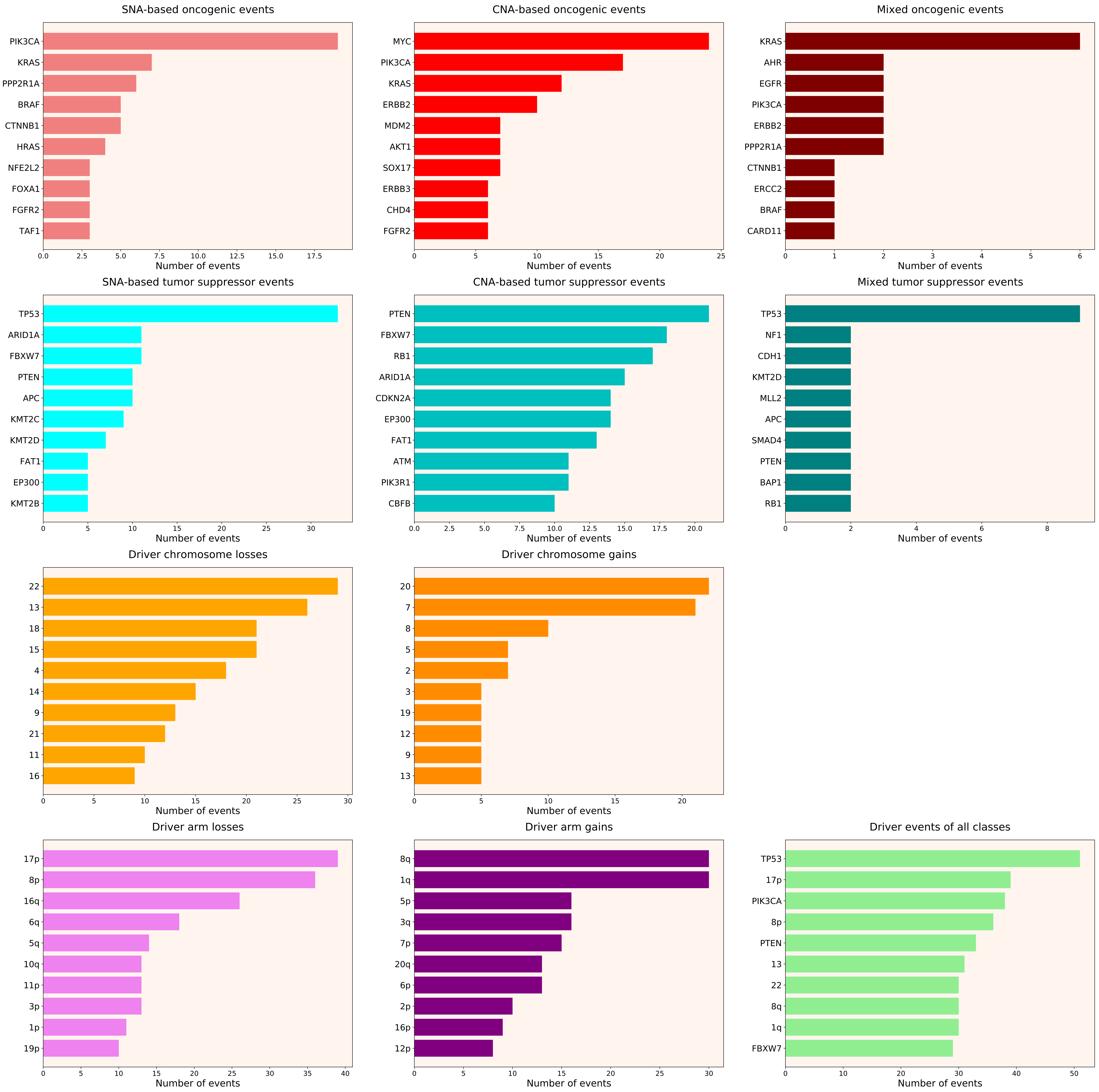
SNA-based oncogenic events
PIK3CA
KRAS
PPP2R1A
BRAF
CTNNB1
HRAS
NFE2L2
FOXA1
FGFR2
TAF1
0.0 2.5 5.0 7.5 10.0 12.5 15.0 17.5
Number of events
CNA-based oncogenic events
MYC
PIK3CA
KRAS
ERBB2
MDM2
AKT1
SOX17
ERBB3
CHD4
FGFR2
0 5 10 15 20 25
Number of events
Mixed oncogenic events
KRAS
AHR
EGFR
PIK3CA
ERBB2
PPP2R1A
CTNNB1
ERCC2
BRAF
CARD11
0 1 2 3 4 5 6
Number of events
SNA-based tumor suppressor events
TP53
ARID1A
FBXW7
PTEN
APC
KMT2C
KMT2D
FAT1
EP300
KMT2B
0 5 10 15 20 25 30
Number of events
CNA-based tumor suppressor events
PTEN
FBXW7
RB1
ARID1A
CDKN2A
EP300
FAT1
ATM
PIK3R1
CBFB
0.0 2.5 5.0 7.5 10.0 12.5 15.0 17.5 20.0
Number of events
Mixed tumor suppressor events
TP53
NF1
CDH1
KMT2D
MLL2
APC
SMAD4
PTEN
BAP1
RB1
0 2 4 6 8
Number of events
Driver chromosome losses
22
13
18
15
4
14
9
21
11
16
0 5 10 15 20 25 30
Number of events
Driver chromosome gains
20
7
8
5
2
3
19
12
9
13
0 5 10 15 20
Number of events
Driver arm losses
17p
8p
16q
6q
5q
10q
11p
3p
1p
19p
0 5 10 15 20 25 30 35 40
Number of events
Driver arm gains
8q
1q
5p
3q
7p
20q
6p
2p
16p
12p
0 5 10 15 20 25 30
Number of events
Driver events of all classes
TP53
17p
PIK3CA
8p
PTEN
13
22
8q
1q
FBXW7
0 10 20 30 40 50
Number of events
